# Optimisation of *Calophyllum inophyllum* seed oil nanoemulsion as a potential wound healing agent

**DOI:** 10.1186/s12906-022-03751-6

**Published:** 2022-11-04

**Authors:** Elnaz Saki, Vinuthaa Murthy, Roshanak Khandanlou, Hao Wang, Johanna Wapling, Richard Weir

**Affiliations:** 1 College of Engineering, IT & Environment, Charles Darwin University, Casuarina campus, Darwin City, 0810 Australia; 2 Menzies School of Health Research, Charles Darwin University, Darwin City, Australia; 3 Berrimah Veterinary Laboratory, Department of Industry, Tourism and Trade Northern Territory Government, Darwin City, Australia

**Keywords:** Nanoemulsion, RSM, Cell viability, Wound healing

## Abstract

**Background:**

Efficient delivery systems of *Calophyllum inophyllum* seed oil (CSO) in the form of nanoemulsion were optimised to enhance its stability and ensure its therapeutic efficiency as a potential agent for various biomedical applications.

**Method:**

Response Surface Methodology (RSM) was used to determine the effects of independent variables (oil, surfactant, water percentage and homogenisation time) on physicochemical characteristics, including droplet size, polydispersity index and turbidity.

**Results:**

The optimised CSO nanoemulsion (CSONE) has a 46.68 nm particle size, 0.15 Polydispersity index value and 1.16 turbidity. After 4 weeks of storage at 5 ± 1 °C and 25 ± 1 °C, the CSONE was physically stable. The optimised CSO nanoemulsion showed enhancement in cell viability and wound healing in baby hamster kidney a clone BHK-21 (BSR) cells as compared to the CSO. The wound healing property of CSONE was higher than CSO.

**Conclusion:**

Thus, our in vitro wound healing results demonstrated that CSO in the nanoemulsion form can promote wound healing by enhancing the proliferation and migration of epidermal cells.

**Graphical Abstract:**

The *coarse emulsion of Calophyllum inophyllum seed oil nano* emulsion was prepared using high shear homogeniser techniques. The optimised CSONE with the droplet size of 46.68 nm was prepared from a mixture of CSO, Tween 80, and high pure water (HPW), then used for the biological investigation. The in vitro cell monolayer scratch assay revealed that CSONE in the lowest concentration of CSO resulted in 100% wound closure after 48 hrs. The optimised CSO nanoemulsion was found to be a promising and effective approach in the treatment of wounds by boosting the proliferation and migration of epidermal cells. 
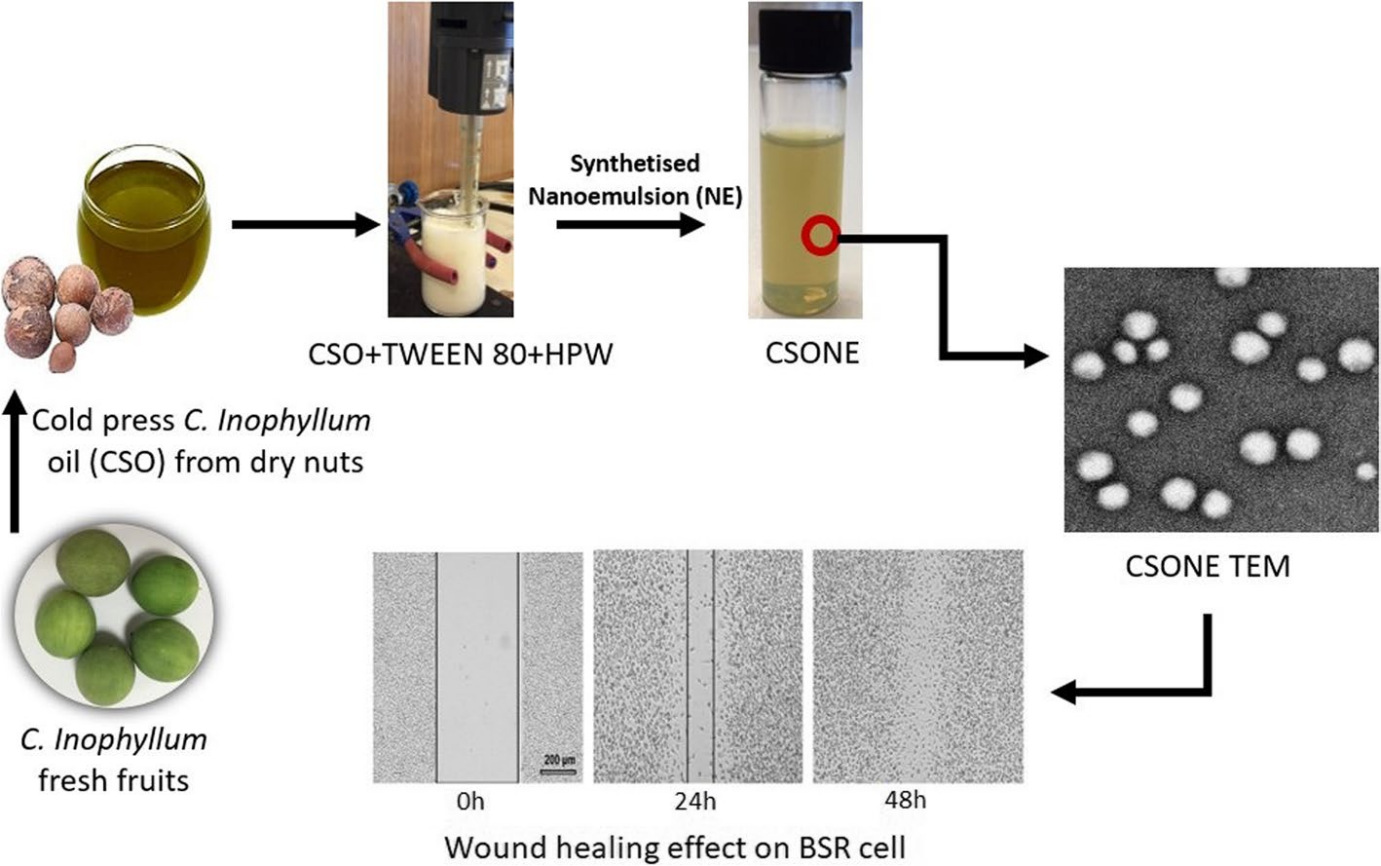

**Supplementary Information:**

The online version contains supplementary material available at 10.1186/s12906-022-03751-6.

## Background

*Calophyllum inophyllum L*. (Clusiaceae), commonly known as Alexandrian laurel or beauty leaf tree, produces round fruit with single-seeded berries and brownish-orange when mature. *C. inophyllum* seed oil (CSO) is nonedible, and studies from India, East Asia and Australia show that it could be used as a source for biodiesel [1–3]. Previous studies on *C. inophyllum* extracts identified potential pharmaceutical values, including anti-HIV, antioxidant, antimicrobial, anti-*Helicobacter pylori*, anti-proliferative, antitumor, antifungal and anti-inflammatory activities [4–9].

Some studies describe the in vitro and in vivo wound healing effects of CSO due to its active compounds, including calophyllolide. In the scratch test assay on human keratinocyte cells, incubation in 0.1% of the oil extract of *Calophyllum inophyllum* accelerated wound closure with the healing factor 1.3 to 2.1 higher than the control [10–12]. Wound healing is amongst the most complicated processes in the human body. The skin presents an efficient barrier to defend the body from the penetration of molecules and microorganisms in the external atmosphere and from extreme water, loss to maintain homeostasis [13]. Skin barrier repair requires the participation of multiple cell types in consecutive steps. Different stages of haemostasis, inflammation, angiogenesis, growth, re-epithelialisation, and remodelling happen in an overlapping but temporary order [13, 14]. The range of substances that are appropriate for diffusion into the Stratum Corneum (SC) to repair the skin barrier are generally restricted to small molecular weight (MW < 500 Da) compounds with balanced lipophilicity (log 1–3) and a measurable solubility in both oil and water, excluding many possibly therapeutic compounds that fall outside these standards [15, 16]. To overcome this, nanosystems could be employed as effective delivery systems to penetrate the skin barrier reaching different skin layers, including the SC and deeper tissues, to achieve efficient wound healing.

Nanosystems include liquid emulsions (microemulsions and nanoemulsions), solid lipid nanoparticles, nanostructured lipid carriers and advanced nanovesicles, such as flexible and ultra-flexible liposomes [15]. These systems can offer enhanced efficacy and targeted delivery and gain bioavailability and stability of the active compounds. Moreover, nanosystems could detract active compounds from irritation and promote the formulation of lipophilic or poorly water-soluble components [17].

Nanoemulsions (NE) are generally oil-in-water emulsions with mean droplet diameters ranging from 50 to 1000 nm, with the average droplet size typically between 100 and 500 nm. The particles may arise as oil-in-water and water-in-oil formulae, where oil or water can be the particle’s core [18]. Infusion into the skin via several mechanisms can be enhanced by nanoemulsions that, firstly, facilitate the solubilisation of hydrophilic and lipophilic compounds, increasing the formulation’s loading capacity and dosage application. Secondly, their large surface area and good skin contact, attached to their occlusive nature, ensure good surface contact with the SC surface. Finally, their oil and surfactant components may have a straight permeation enhancement effect on the SC lipid structure [15]. Nanoemulsions also have better stability in response to flocculation, sedimentation, and creaming than conventional oil [19].

The traditional pharmaceutic formulation of any dosage figure involving trial and error methodology is time-consuming, costly, and inconsistent. The design of experiments (DoE) has been suggested as a statistical optimisation method for studying the interaction between independent and dependent variables. The DoE provides the most meaningful information and cooperation between variables in the minimum number of runs. Response surface methodology (RSM) is a standard DoE frequently used to evaluate the response surface’s main effect, interplay, and shape [20]. Several RSM designs, such as Box–Behnken, factorial design, central composite design and optimal combined design (OCD), are available for multivariable formulation optimisation. The OCD is the part of the RSM space where multivariate combinations of mixture components (MCs) and process variables (PVs) have been expressed to display certain quality.

The objective of the present study is to develop the best formulation of CSO loaded nanoemulsion for epidermal delivery. Optimisation of formulation requires the identification of the nanoemulsion variables affecting its characteristics, including particle size, polydispersity index, turbidity and stability evaluation. By encapsulating the CSO into a nanoemulsion, it could offer better bioavailability, antioxidant and wound healing activity.

## Methods

### Chemicals and reagents

Polysorbate 80 (Tween 80) (P6224), sodium methoxide (S0485), n-hexane (270504), fatty acid methyl ester (FAME) standard (CRM47885), Formazin 4000 NTU (TURB4000), 3-(4,5-dimethylthiazol-2-yl)-2,5-diphenyl tetrazolium bromide (MTT) (M2128) and dimethyl sulfoxide (DMSO)(D8418) were purchased from Sigma Aldrich, Australia and 2,2-diphenyl-1-picryl-hydrazyl (DPPH) (ALF044150), butylated hydroxytoluene (BHT) (ACR11299) were purchased from Thermo Fisher Scientific, Australia.

### Fatty acid analysis by gas chromatography-mass spectrometry

*Calophyllum inophyllum* seed oil was purchased from a Darwin-based company that produces the oil in bulk using a cold press method from seeds collected in the Darwin region of the Northern Territory (NT), Australia. Samples of CSO were derivatised to fatty acid methyl ester [21]. Briefly, 100 μl of CSO was mixed with 5 mL of sodium methoxide in methanol (0.5 N) in a screw cap test tube and heated in a water bath at 50 °C for 20 min. The test tube was cooled to room temperature before adding n-hexane (5 mL). The mixture was shaken for 5 min and then centrifuged at 1750 rpm for 5 min. The upper layer of the FAME sample was injected into a gas chromatography-mass spectrometry (GC-MS; Agilent 5975C GC/MS system with a DB-5 (30 m × 0.25 mm × 0.25 μm) ms capillary column coated with polysiloxane for analysis. The carrier gas was helium at a 1.5 mL/min constant flow rate. The initial temperature of the column was 70 °C and raised to 300 °C at 9 °C/min and then maintained for 4 minutes at 300 °C. A diluted FAME standard in hexane was used as a reference.

### Neoflavonoids analysis by liquid chromatography/mass spectrometry

To check the presence of the neoflavonoids, the CSO was washed with 90% MeOH at a 1:1 ratio v/v. The MeOH layer was collected and submitted to chromatographic analysis using an LC-QTOF/MS system (Agilent Technologies), consisting of a 1260 LC coupled to a 6530 QTOF-MS equipped with an electrospray ionisation source. LC separation was accomplished on a Zorbax Eclipse plus C18 column (2.1× 50 mm, 1.9 μm) at 35 °C. The injection volume was 2.0 μL. The mobile phases consist of solution A 0.1% v/v formic acid in water and solution B 0.1% formic acid in acetonitrile. The MS was tuned for a low mass range (up to 1700 m/z) and run positive mode for a full scan. Data were collected and processed using “MassHunter” B.05.00 Service Pack 3.

### Preparation of *Calophyllum inophyllum* seed oil nanoemulsion

The CSONE was prepared by mixing a dispersed phase and a continuous phase. The CSO was dissolved using Tween 80 as a surfactant in the dispersed phase. The aqueous phase consisted of high pure water. A coarse oil-in-water (O/W) type emulsions were first prepared [22, 23] by mixing the oil and water phases at 200–300 rpm for 15 min at 40 °C using an overhead stirrer. For nanoemulsion preparation, the coarse emulsion was homogenised at 20000 rpm for 20 minutes using a high shear homogeniser (IKA T18 Ultra-Turrax, Germany). The emulsion temperature was controlled at < 40 °C by placing it in an ice bath during homogenisation. The nanoemulsion was divided into two test tubes and stored in the refrigerator at a temperature of 5 ± 1 °C and a room temperature of 25 ± 1 °C, respectively, for further analysis.

### Particle size and polydispersity index analysis

The particle size and PDI of the CSONE were measured with the Nanoparticle Analyzer SZ-100 (HORIBA, Japan) using dynamic light scattering, scattered at an angle of 173° and a temperature of 25 °C. The mean hydrodynamic diameter (z-average mean) was calculated from the autocorrelation function of the intensity of light scattered from the particles. Each sample was diluted with deionised water at a 1:100 ratio to avoid the inter particulate interaction and multiple scattering [24]. The droplet size and PDI measurements were conducted in triplicates.

### Turbidity measurement

The turbidity of the samples was evaluated using a UV–Vis spectrophotometer (Varian, Cary 100) at 600 nm. The turbidity measurement is a simple and low-cost method of defining the stability of an emulsion [25]. For each measurement, samples were diluted with deionised water at a 1:100 ratio. The measurement was performed at 25 °C. A standard curve was plotted using Formazin 4000 NTU as a turbidity calibration standard.

### Optimisation of CSONE formula by RSM and statistical analysis

Nanoemulsion formulation was optimised according to a novel Optimal Combined Design (OCD) with three component factors (CSO, Tween 80 and HPW) and a numeric factor (homogenisation time). A total of 24 runs were generated using Design-Expert®12 software (Stat ease Inc., Minneapolis, USA). Optimal Combined Design is a flexible design structure to accommodate custom models, categoric factors, and irregular (constrained) regions. It was used to study the effects of the independent variables: CSO (X_1_), Tween 80 (X_2_), high pure water (HPW) (X_3_) and homogenisation time (X_4_) on the responses: droplet size, PDI and turbidity of CSO nanoemulsions. In the current design, three mixture components (Table S1 in Supporting Documentation) and component factors were evaluated by changing individual concentrations while keeping their total concentration constant with a sum of 100.

1 $${X}_1+{X}_2+{X}_3=100\%$$

For constructing the final model, a nonlinear response function was required. Response Surface Methodology presents crucial knowledge on the impact of variables and responses of interest with the smallest number of experiments. A second-order quadratic equation was used to express the responses: droplet size, PDI and turbidity of CSO nanoemulsions as a function of the independent variables as follows:

2 $$Y={\beta}_0+{\sum}_{i=1}^k{\beta}_i{X}_i+{\sum}_{i=1}^k{\beta}_{ii}{X}_i^2+{\sum}_{i=1}^k{\sum}_{j=1}^k{\beta}_{ij}{X}_{ij}{X}_{ji}+\varepsilon$$

Equation 2 models the response Y with the factors of independent variables X. This model incorporates the models of complex orders, where *k* represents the total number of patterns of a particular order and *i* and *j* represent individual patterns. The coefficients for various orders are referred to as β, where β_*ij*_ represents the interaction effect, β_*ii*_ for the quadratic effect, and β_0_ represents the final adjustment constant. The error between the observed and predicted values is represented by the term ε in the model [26].

### Transmission electron microscope analysis of nanoemulsion

The morphology and nanostructure of the CSONE formulation were evaluated using a JEOL 1400 Transmission electron microscope (TEM) at 120 kV. Briefly, 50 μL of the nanoemulsion was diluted 1:100 with deionised water and filtered through a 0.22 μm filter membrane. Then samples were cast mounted onto 200 mesh formvar coated copper grids for a minute and stained with a 2% solution of uranyl acetate (UA). The excess liquid and prepared samples were dried using the Whatman filter paper at room temperature [27]..

### Stability study of CSONE

Freshly prepared samples were put in a container and stored in a refrigerator with a temperature of 5 ± 1 °C and at a room temperature of 25 ± 1 °C for 4 weeks. To test the physical stability of the samples, they were centrifuged at 4500 rpm for 15 minutes and then observed for layer separation. The particle size and PDI values were analysed before and after 4 weeks of storage.

### Cell viability assay of CSONE

Cytotoxicity of CSO and CSONE against baby hamster kidney clone BHK-21 (BSR) cells was determined by the MTT assay [28]. The BSR cells, which are adherent cell line used in molecular biology, was obtained from Berrimah Veterinary Laboratory, NT, Australia. Due to the nonsolubility of CSO in an aqueous medium, the stock solution was dissolved in DMSO. The maximum DMSO concentration was 0.4% in the medium. Briefly, 150 𝜇L of 1 × 10^5^ cells were seeded a 96 well plate in complete Gibco Dulbecco’s modified Eagle’s medium with supplements (DMEM) with 5% foetal bovine serum and 1% antibiotic/antimitotic (100 units of peniciline, 0.1 mg of Streptomycine and 0.25 μg of Amphotericin) in a final volume of 300 μL. Following 72 hours of incubation, the cells were confluent, and 150 𝜇L fresh media was replaced before adding the samples. Then, 150 𝜇L of CSONE, CSO, DMSO, Tween 80 and HPW was added in triplicate wells. The final concentrations of CSO were in the same range as optimised CSONE, from 73 to 2.28 μg/mL. The Tween 80 concentration used as a control was 2.6 times greater than the CSO concentration in the nanoemulsion. The microplates were incubated for 24 hours at 37 °C in an air-conditioned environment of 5% CO_2_.

After the samples were incubated for 24 hours, 20 μL of 5 mg/mL of MTT was added to each well and incubated for an additional 4 hours. Active mitochondria in live cells reduced MTT to crystalline purple-blue formazan. The number of living cells was proportionate to the amount of crystalline purple-blue formazan produced. After incubation, media in each well was discarded and 100 *μ*L of 10% sodium dodecyl sulphate (SDS) solution was added to solubilise the purple-blue formazan. The absorbance was measured with an ELISA microplate reader (xMark™, BIO-RAD, Australia) at 570 nm. A graph of the percentage of cell viability versus concentration of samples was plotted, and the IC50 (concentration that inhibits 50% of cell growth compared to control) of cells were calculated using

3 $$\% cell\ viability=\Big[\ {}^{{Abs}_{sample}}\!\left/ \!{}_{{Abs}_{control}}\Big]\right.\times 100$$

where, Abs _control_ is the absorbance of untreated cells and Abs _sample_ is the absorbance of treated cells with samples.

### The antioxidant activity of CSONE

The antioxidative capacity comparison of CSO and CSONE was evaluated using the stable DPPH radical method. This spectrophotometric assay uses stable free radical DPPH as a reagent. In this assay, BHT was used as a positive control. Fifty microliters of CSONE or CSO in methanol over the range of 15.6–250 μg/mL were added to 5 mL of a 0.04% methanol solution of DPPH. These concentrations were selected based on previous studies on NE formulation [29, 30]. After a 30 min incubation at room temperature, the absorbance was read against a blank at 517 nm with an ELISA microplate reader (xMark™, BIO-RAD, Australia). Tests were carried out in triplicate. Inhibition of free radical DPPH in percentage was calculated using the following equation.

4 $$Total\ antioxidant\ activity\%={}^{ Abs- Abs\ sample}\!\left/ \!{}_{ Abs\ control}\right.\times 100$$

### Evaluation of the wound healing effect of CSONE and CSO

The scratch assay evaluated the in vitro wound closure effect on BSR cells [31]. The fibroblast growth factors (FGF) used in this study as a positive control growth factor are a family of cell signalling proteins involved in various processes, most notably for normal development [32]. Briefly, 150 μL of 1 × 10^5^ cells were seeded to 4-well chamber slides and incubated with complete Gibco Dulbecco’s modified Eagle’s medium with supplements (DMEM) with 5% foetal bovine serum and 1% antibiotic/antimitotic (100 units of peniciline, 0.1 mg of Streptomycine and 0.25 μg of Amphotericin) at 37 °C and 5% CO_2_. After a 72 h incubation, the confluent monolayer of cells was scratched horizontally with a sterile P100 pipette tip. The medium was discarded, the cells were washed with PBS, and 150 μL of fresh medium was added to each well. Then, cells were treated by adding 150 *μ*L of CSO or CSONE with a final concentration of 9.12 *μ*g/mL and 4.56 *μ*g/mL to a final volume of 300 *μ*L. The cells without treatment (distilled water added to the cell with media) and Fibroblast Growth Factors to a final concentration of 2 ng/mL (FGF; Sigma Aldrich, Germany) were used as the control and positive control, respectively. Wound closure was monitored using an OLYMPUS DP22 Microscope Digital Camera, with cell migration evaluated using image analysis.

Wound closure can be calculated by measuring the decrease of an uncovered area at diverse time points until the ‘wound’ is closed [33]. Three images were taken from the scratched area in each chamber. These areas were compared at times T_0_, T_1_ and T_2_ to determine the percentage of wound closure. The experiments were performed in triplicate, and the data were recorded, with wound closure percentages calculated using

5 $$Wound\ Closure\%=\left[{}^{{A}_{T= oh}-{A}_{T=\Delta h}}\!\left/ \!{}_{{A}_{T= oh}}\right.\right]\times 100$$

where, A_T = 0h_ is the distance of wound measured immediately after scratching made, and A_T = ∆h_ is the distance of wound measured h hours after scratching made.

### Statistical analysis

Experimental data were statistically analysed using Design Expert Software (version 12.0.3.0; Stat-Ease, Minneapolis, USA). To select the best fitting polynomial model, numerous statistical parameters (lack-of-fit, predicted and adjusted multiple correlation coefficients and coefficient of variation) of different polynomial models were compared. The significant difference was determined through analysis of variance by calculating F-value at the probability of 0.5, 0.1 and 0.01. All these experiments were performed in triplicate. The results obtained from the biological experiment were performed three times and were analysed as mean ± 2SD. Statistical significance was determined using a two-way ANOVA. *P* < 0.05 was considered to be statistically significant.

## Results

### Characterisation of CSO fatty acid and neoflavonoids

**Fig. 1:**
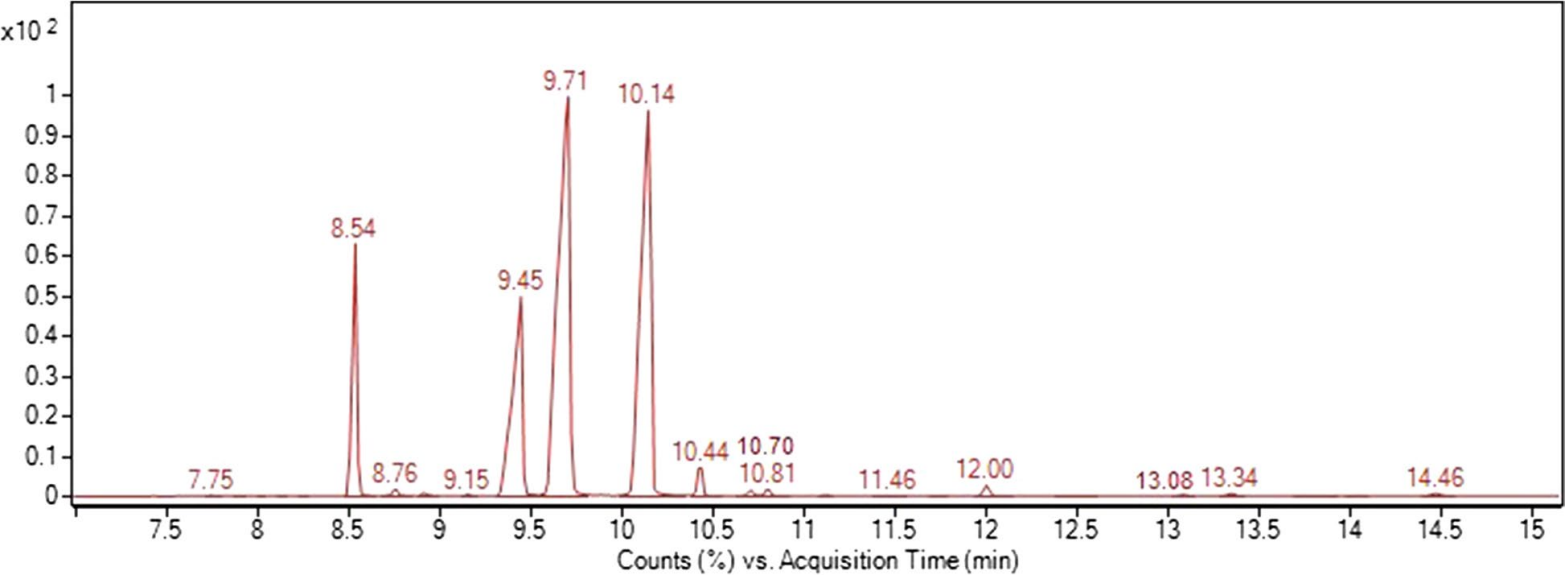
Gas chromatography-mass spectrometry (GC-MS) chromatogram of fatty acids of *Calophyllum inophyllum* seed oil (CSO)

Table 1 reports the main fatty acids identified in *Calophyllum inophyllum* seed oil n-hexane extract using Gas chromatography-mass spectrometry. A total of 15 compounds were identified (Fig. 1). Oleic acid (38.30%) was the most abundant, followed by Linoleic acid (32.37%). The neoflavonoids were identified from CSO using Liquid chromatography/mass spectrometry (LC-MS) by comparing the major ion fragment data with previous works. The identified constituents were Calophylloide, inophyllum D, inophyllum P, inophyllum C, tamanolide, tamanolide D, tamanolide P, 12-Oxo-calanolide, calanolide A.

**Table 1:** The main fatty acids of *Calophyllum inophyllum* seed oil CSO from Gas chromatography-mass spectrometry (GC-MS)

No.	Retention Time (min.)	Area (%)	Fatty acids
1	7.75	0.02	Myristic acid
2	8.54	9.86	Palmitic acid
3	8.76	0.31	Palmitoleic acid
4	9.15	0.15	Margaric acid
5	9.45	16.09	Stearic acid
6	9.71	38.30	Oleic acid
7	10.14	32.37	Linoleic acid
8	10.44	1.29	Arachidic acid
9	10.70	0.24	α-Linolenic acid
10	10.81	0.31	11-eicosenoic acid
11	11.46	0.04	Linolenic acid
12	12.00	0.51	Behenic acid
13	13.08	0.06	Tricosanoic acid
14	13.34	0.14	Eicosatrienoic acid
15	14.46	0.18	Lignoceric acid

### Optimisation of CSONE using optimal combined design

The response surface models can explain the variation in the response variables studied as a function of main emulsion components. The mixture components are interconnected as they are compelled to sum to 100, though the procedure variables can be altered independently. The CSONE was prepared using diverse levels of independent variables. The high shear homogeniser speed rate was kept constant at 20000 rpm for all experiments. The droplet size, PDI and turbidity of CSONE obtained from the experiments are presented in Table S2 in the Supporting Documentation.

### Effect of independent variables on response variables

The final model obtained to predict the droplet size, PDI value and turbidity of CSONE in terms of the actual factors of mixture components can be expressed by the following quadratic equations

6 $$\textrm{Size}=264.463\textrm{A}-15.93\textrm{B}+127.628\textrm{C}+12.066\textrm{ABD}-272.266\textrm{ACD}+80.939\textrm{BCD}\ 8.218\textrm{AD}2+34.616{\textrm{BD}}^2$$

7 $$PDI=-317.738A+0.69B+0.849C+526.403 AB+530.732 AC-1421.19 AD-1.032 BC-0.759 BD-0.31 CD-440.138 ABC+233 AB D+2330.96 ACD+5.075 BCD+221.67\ AB\left(A-B\right)+223.151 AC\left(A-C\right)-1.42894\ BC\left(B-C\right)-1907.14 ABCD+941.118 ABD\left(A-B\right)+941.022 AC D\left(A-C\right)+1.1415 BCD\left(B-C\right)$$

8 $$Turbidity=67635.7A+440.529B+2593.76C-82274.6\ AB-69513.2 AC-5034.96\ BC$$

where, A is CSO, B is Tween 80, C is HPW and D is homogenisation time.

Statistical analysis of the models was obtained and listed in Table 2, which summarises the R^2^ values, lack of fit *P*-value and model *P*-value. The most suitable model was fitted based on the following criteria: low *P*-value (< 0.05), insignificant lack of fit, high R^2^ (> 0.90), low standard deviation, a randomly scatter plot of residuals, and whether it can predict the validation set.

**Table 2:** Analysis of variance (ANOVA) for the Optimal Combined Design (OCD)

Source / Parameters	Size	PDI	Turbidity
R^2^	0.9213	0.9981	0.9027
Adjusted R^2^	0.8756	0.9890	0.8756
Predicted R^2^	0.8605	0.7961	0.8366
Lack of fit	0.0755	0.0996	0.0772
*p*-value	Not significant	Not significant	Not significant
Model	0.0001	0.0002	0.0001
*p*-value	Significant	Significant	Significant

### Droplet size

Analysis of variance (ANOVA) for the droplet size (Table 2) determined that the resultant quadratic patterns well described the experimental data with the coefficients of various determinations, R^2^ of 0.921. The quadratic model also showed that the predicted R^2^ of 0.8605 reasonably agrees with the adjusted R^2^ of 0.8869 (Eq. 6). Furthermore, the quadratic term of size had a significant effect (*P* < 0.05) on the nanoemulsions’ droplet size.

### Polydispersity value

The PDI value ANOVA indicated that the resultant cubic linear model (Eq. 7) adequately described the experimental data with the coefficients of multiple determinations (R^2^) of 0.796 (Table 2). The lack of fit was also non-significant (*P* ≤ 0.05) related to the net error for all variables, which expressed that the model is statistically precise regarding the PDI value.

### Turbidity

The turbidity ANOVA (Eq. 8) showed that the turbidity of CSONE could be predicted by a quadratic polynomial model, R^2^ of 0.9027. The model also showed that the predicted R^2^ of 0.8366 was in reasonable agreement with the adjusted R^2^ of 0.8756. Furthermore, the quadratic term of turbidity had a significant effect (*P* < 0.0001) on the nanoemulsions’ turbidity. The Turbidity values are expressed as NTU (Nephelometric Turbidity Units), with the turbidity feature depending on numerous parameters, such as droplet concentration and PDI.

**Fig. 2:**
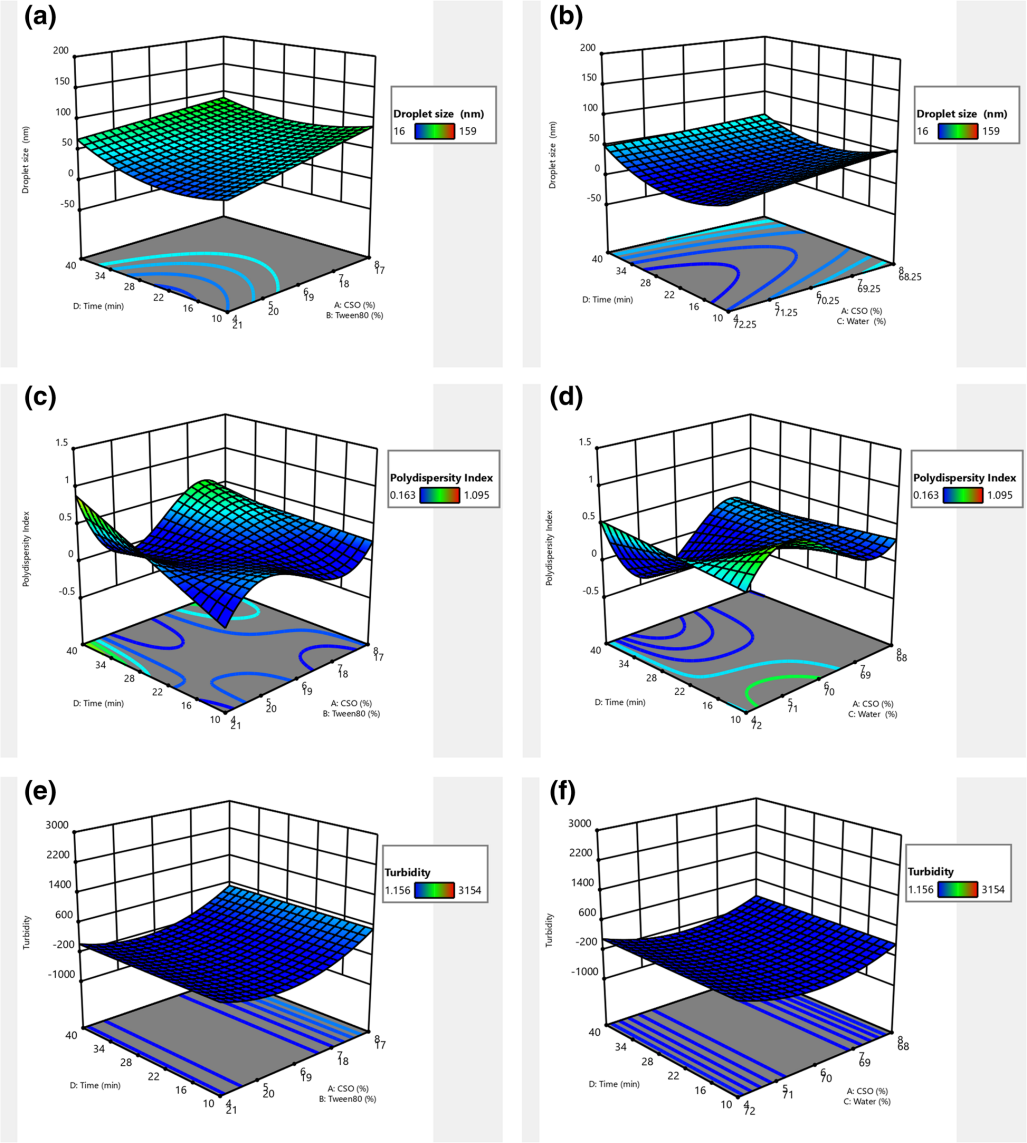
Response surface plots showing the interaction effect between three variables. Droplet size (**a**–**b**), Polydispersity index (**c**–**d**) and Turbidity (**e**–**f**)

The correlation between variables are further described using the 3D surface plots. Figure 2 (a–f) shows the effect of factors A, B, C and D on droplet size, PDI and turbidity. The correlation between experimental and predicted values using the developed model for droplet size (A), PDI value (B) and turbidity (C) also indicates a good agreement between the actual and predicted responses (Fig. S1 in the Supplementary Materials).

### Verification of the CSONE optimisation results

Optimised emulsifying conditions were used to check the competency of the model for the prediction of response values. For this purpose, the response surface methodology, using a mixture design, was adopted for finding optimal conditions. The optimum parameters for preparation of CSONE were 7.31% of CSO, 19.39% of Tween 80, 73.28% of HPW and 20 min homogenisation time. Thus prepared, the CSONE gave a particle size of 46.68 nm, PDI value 0.19 and turbidity 5.49. The experimental values were determined to be close to the predicted value of 52.43 nm particle size, 0.15 PDI value and 1.16 the turbidity of CSONE, with 0.57 desirability. The visual appearance of nanoemulsion was clear and transparent, indicating a stable nanoemulsion [34] (Fig. S2 in Supplementary Materials).

### Transmission electron microscopy observation of CSONE droplets

**Fig. 3:**
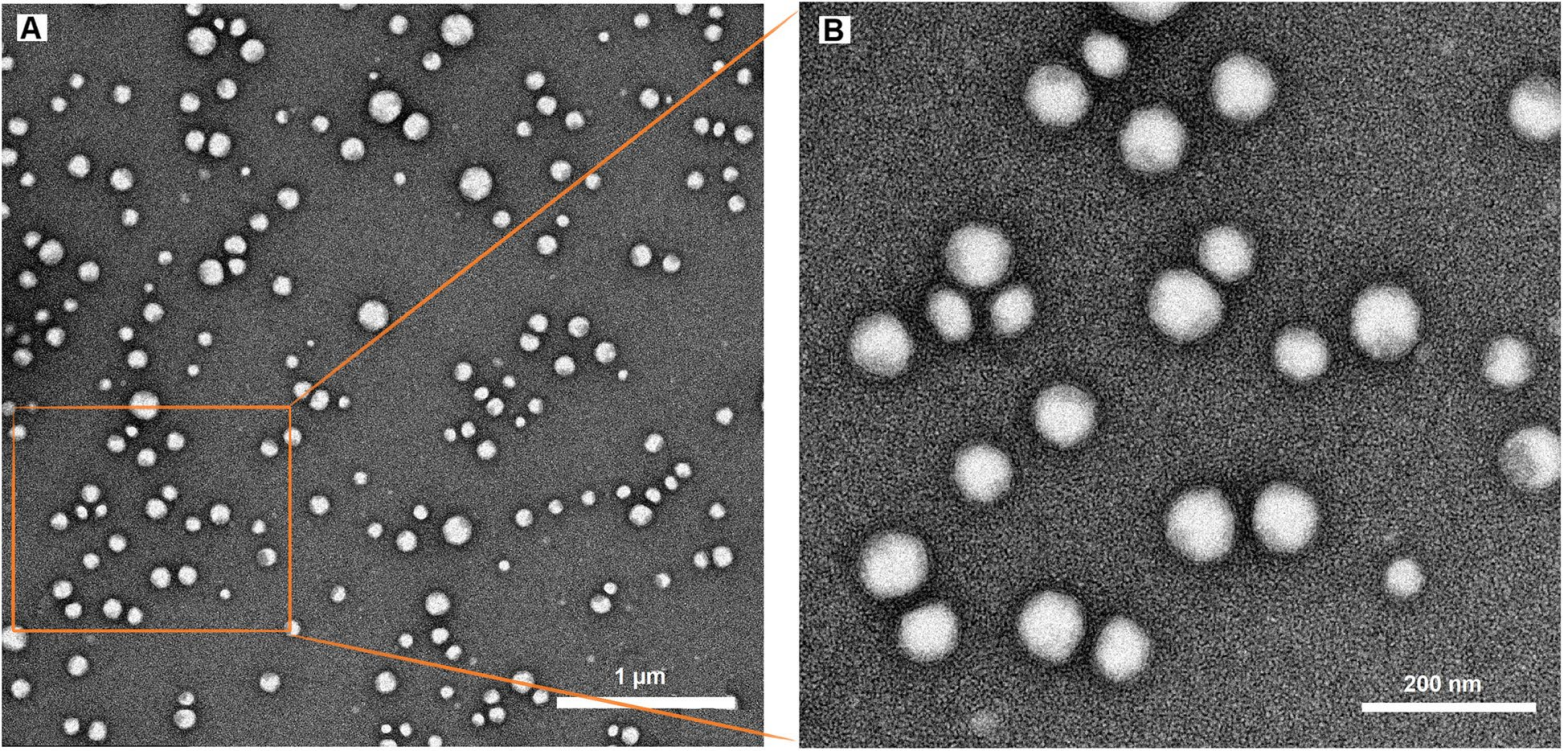
Transmission electron microscopy of droplets in the optimised *Calophyllum inophyllum* seed oil nanoemulsion (CSONE). Scale bar. A: 1 μm; B: 200 nm

Transmission electron microscopy was used to observe the morphology of CSONE. Figure 3 (a–b) shows the UA-stained CSONE droplets, with 1 μm and 200 nm scale bars. The micrograph confirmed the spherical shape of CSONE droplets with no aggregation; the grey parts of the droplet were CSO combined in the emulsion system. The average size of the CSONE from the TEM imaging was in the range of 30–60 nm, which is in good agreement with the values obtained by DLS measurement.

### Stability study of CSONE

Every nanoemulsion formulation must have enough physical stability. To evaluate the physical stability of a nanoemulsion, its size and phase separation was tested after 4 weeks of storage. The optimised nanoemulsion sample was found to be physically stable at 5 ± 1 °C and 25 ± 1 °C, following storage.

### Cell viability assay of CSONE

**Fig. 4:**
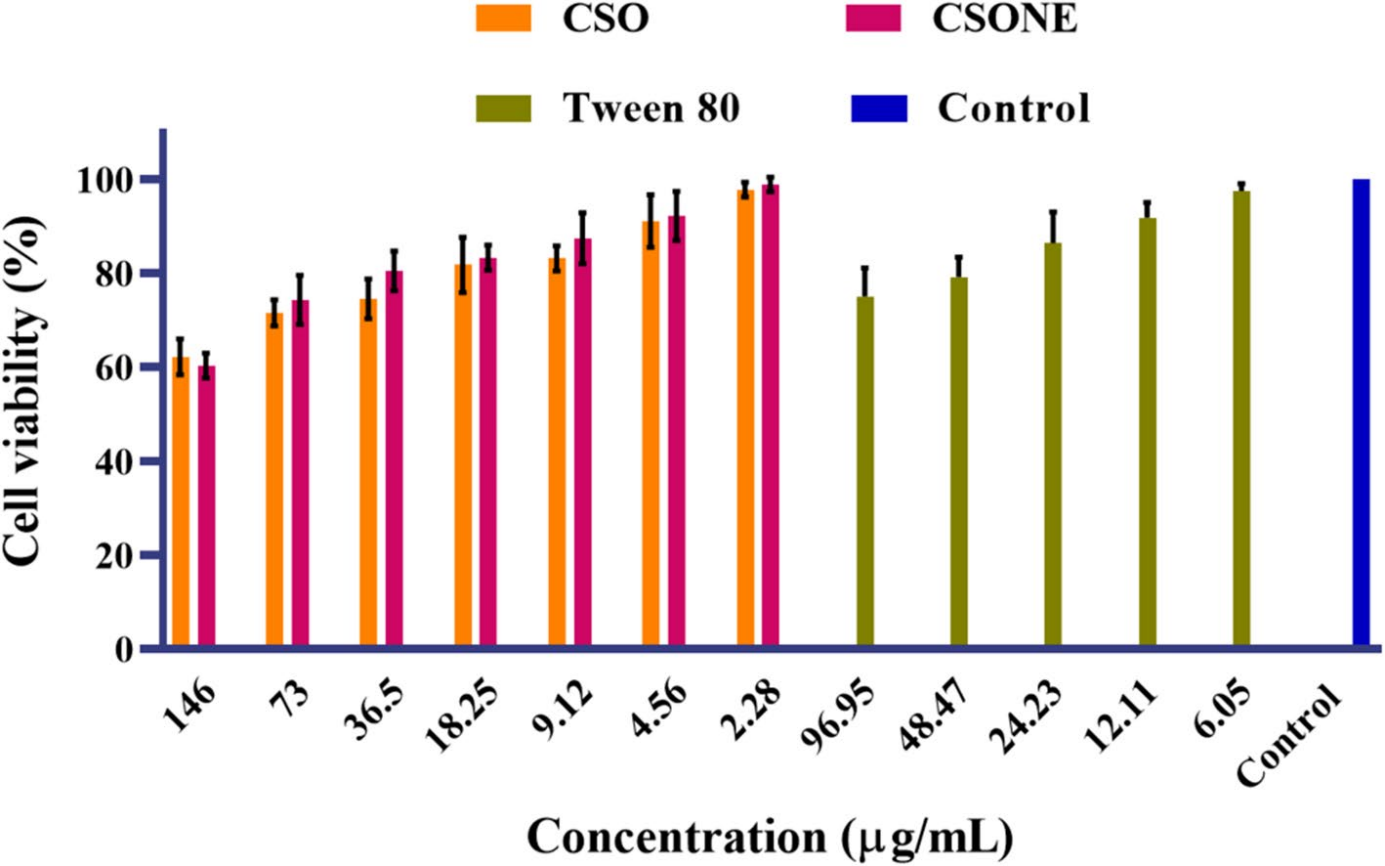
Cell Viability by MTT assay of CSO and CSONE at 24 hr., on BSR cells. The results are against the control of 100% cell viability. MTT (3-(4,5-dimethylthiazol-2-yl)-2,5-diphenyl tetrazolium bromide). *Calophyllum inophyllum* seed oil (CSO). CSO nanoemulsion (CSONE). The Tween 80 concentration used as a control was 2.6 times greater than the CSO concentration available in the nanoemulsion. Each value is the mean of three replicates with standard deviation (± 2SD) and analysed by Two-way-ANOVA (*n* = 3; *p* < 0.01)

The cell viability assay of CSO and CSONE was carried out on BSR cells. Cell lines were incubated with different concentrations of CSO, CSONE and Tween 80. An equivalent amount of Tween 80 present in the NE was also checked for its effect on cell viability, separately. The result showed that after 24 hrs, cell viability increased as the CSO and CSONE concentration decreased, which means that the cell viability of CSO and CSONE was concentration dependent. As shown in Fig. 4, CSO and CSONE did not show significant cytotoxicity and most cell viability percentages were above 70%. Using different concentrations of Tween 80 (6.05–96.95 μg/mL, equivalent to 0.60–9.69%) the results did not show toxic effects and all cell viability values were greater than 75%.

### Free radical scavenging of CSONE

**Fig. 5:**
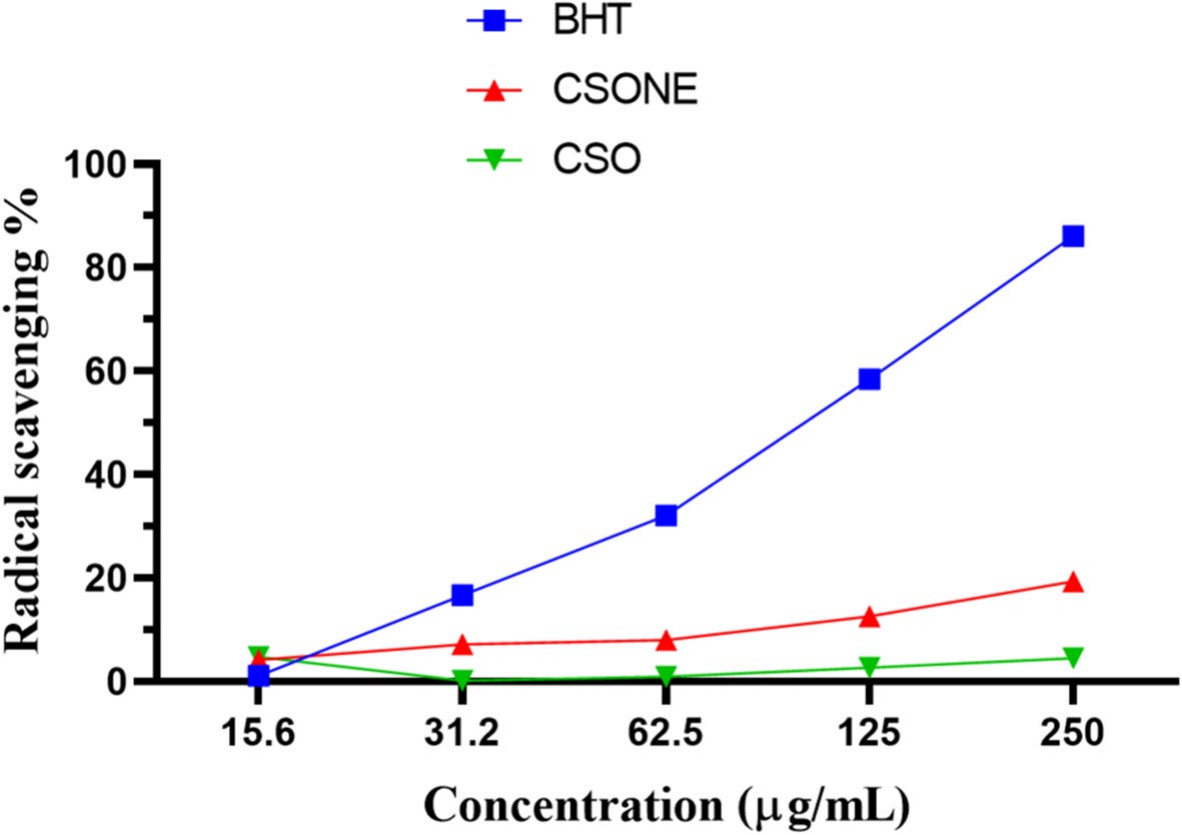
DPPH radical scavenging of CSO, CSONE and butylated hydroxytoluene (BHT). The DPPH results present as Mean ± 2SD

The DPPH radical scavenging ability of CSO and CSONE compared with BHT as a common synthetic antioxidant is shown in Fig. 5. The antioxidant activity of CSO was increased by changing the CSO to a nanoemulsions system. The reduction in droplet size due to nanoemulsion formation aimed to increase the specific surface of CSO such that efficient free radical absorption would be achieved.

### Evaluation of the wound healing effect of CSONE and CSO

**Fig. 6:**
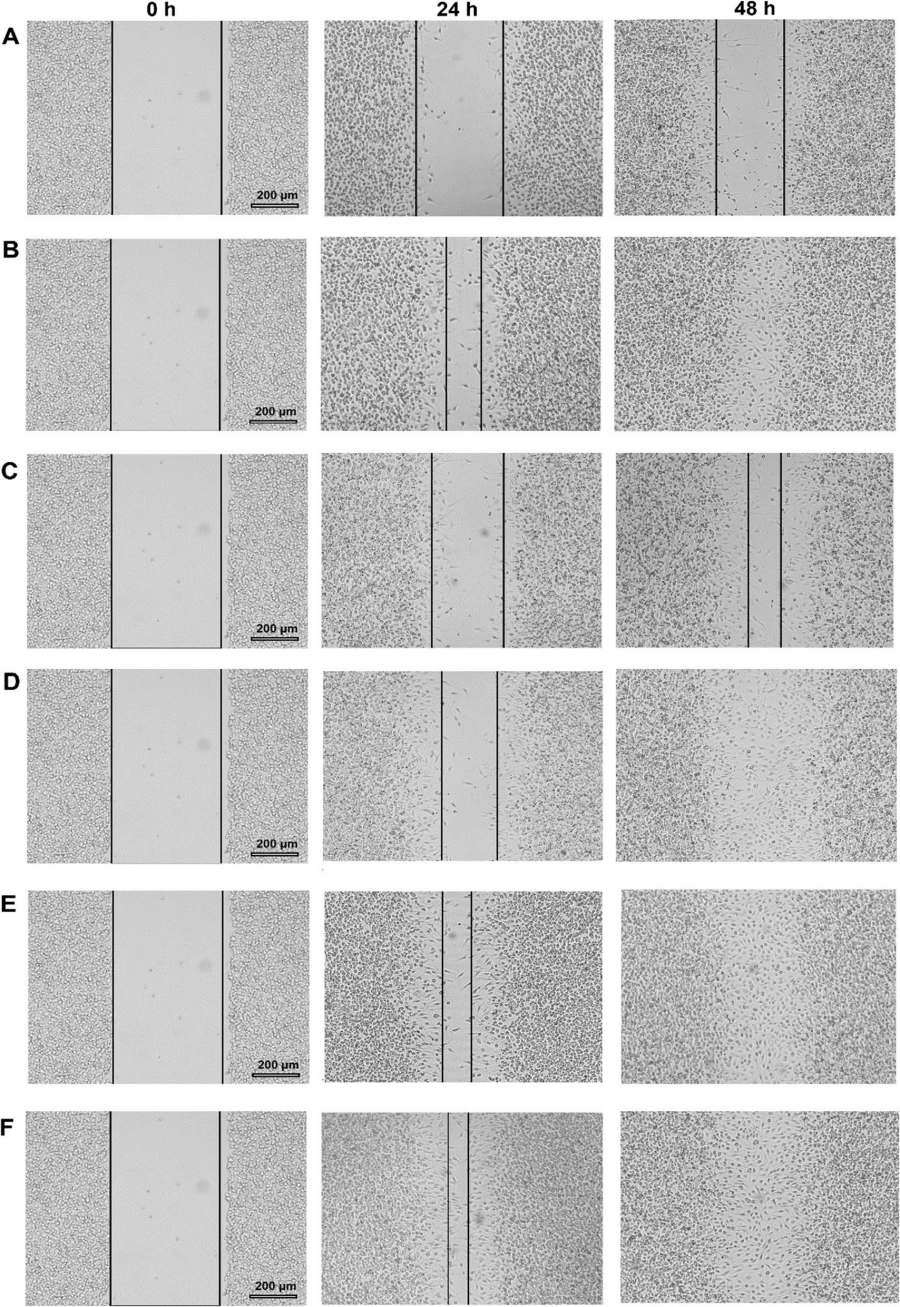
Wound closure photomicrograph of CSO and CSONE at 24 hr. and 48 hr. in BSR cells. *Calophyllum inophyllum* seed oil (CSO) and CSO nanoemulsion (CSONE). **A** Untreated control. **B** Fibroblast Growth Factors (FGF; positive control). **C** CSO1: 9.12 μg/mL.) CSO2: 4.56 μg/mL. (E) CSONE1: 9.12 μg/mL. **F** CSONE2: 4.56 μg/mL. Black solid lines represent the wound size (μm in length) of the BSR cell

In vitro wound healing assay was done to study the coordinated movement of a cell population. The potential wound closure properties of CSONE and CSO were assessed using a monolayer scratch model. Photomicrographs of an optical microscope at 0 hr., 24 hr., and 48 hr. were labelled; shown in Fig. 6 A–F. After 24 hrs exposure to the test samples, it was noted that the cells migrated to the interim gap (Fig. 6 A–F).

The value of wound closure percentage of CSO and CSONE by scratch assay at 12 hr. and 24 hr. in BSR cells are plotted in Fig. S3 in the Supplementary Materials. These results show that the cell contraction was increased until 48 hr. in the test samples. The migration analysis shows that the treated cells with 9.12 μg/mL and 4.56 μg/ml concentration of CSONE resulted in a significant increase in migration with the value of 100% closure, as compared with the untreated cells, which had only 39% closure after 48 hours. In addition, the cells treated with 4.56 μg/mL CSO showed a 100% wound closure. However, cell exposure with 9.12 μg/mL CSO showed only 80% wound closure after 48 hrs, while CSONE exhibited 100% closure. The control groups indicated the natural rate of migration of cells without the influence of any samples or growth factors. Natural wound closure was noted to be the lowest. Fibroblast Growth Factors was used as a positive control and showed complete cell migration after 48 hours.

## Discussion

The chemical analysis of *Calophyllum inophyllum* seed oil from previous studies showed that CSO from different geographical locations has various amounts of fatty acids. Also, the quality of seeds and growth conditions can change the percentage and types of fatty acids [35]. A previous study showed the five tested CSOs from Indonesia, Tahiti, Fiji Islands and New Caledonia contain high amounts of Palmitic acid, Stearic acid, Oleic acid, and Linoleic acid [11], which closely matched our results. As seen in previous studies, the major neoflavonoid in CSO was calophyllolide [9, 10, 12, 36, 37].

The experimental data were used to determine the quadratic polynomial equation. The R^2^ value for turbidity, average droplet size, and PDI were 0.9213, 0.9981 and 0.9027, respectively. If the value of R^2^ is closer to unity, this is an indication of a model better fitting to actual data [38]. The importance of the regression coefficients of a polynomial equation’s regression coefficients is frequently examined by *P*-values [38]. The smaller the *P*-value, the higher the significance of the corresponding factor (Table 2). The factor assessment and the corresponding *P*-values revealed that four independent variables, A) CSO, B) Tween 80, C) HPW, and D) homogenisation time had a remarkable effect on the encapsulation of nanoemulsions (*P* < 0.05).

The droplet size of CSONE depended on Tween 80, CSO, HPW percentage, and homogenisation time at the quadratic level (*p* < 0.0001). Additionally, the homogenisation time was dependent on the mixture components. For example, while the volume of Tween 80 was higher than 19% w/w and HPW 70% w/w, homogenisation time between 15 to 20 min represented the smallest size (Fig. 2a–b). Figure 2a shows that at higher Tween 80 concentrations, a decrease in droplet size of nanoemulsions was detected. Also, CSONE droplet size increased at a high level of HPW, where the CSO level was low, irrespective of time (Fig. 2b). This increase in size was observed due to insufficient emulsifier to cover the smaller newly developed droplets, which begin the coagulation action. At the lower concentration of CSO, surfactant molecules are enough to cover the oil droplets and can reduce interfacial tension at the O/W interface [39].

The mean droplet size is one of the most critical characteristics of emulsions, considering it defines its type (microemulsion or nanoemulsion), features and properties. Moreover, it has been determined that emulsions with large droplet size are more prone to gravity separation or creaming [40]. This study conducted the mean droplet size analyses to develop the best nanoemulsion composition with the smallest droplet size. In this regard, all the prepared emulsions (Table S2 in the Supplementary Materials), were defined as nanoemulsions since the mean droplet size was under 200 nm.

In this study, the main goal was to have a homogenous CSONE system with the lowest PDI values, or in other words, a monodisperse system. The PDI value of CSONE was dependent on Tween 80:CSO as well as CSO:HPW ratio due to its significant effect on droplet size at linear term (*p* < 0.0002). The results indicate that the PDI of the nanoemulsion systems was decreased by increasing the time of the homogenisation process, where the most uniform and homogeneous systems (with a lower PDI) were obtained by homogenisation times greater than 15 minutes. The literature found that an increase in homogenisation time could increase the efficiency of encapsulation of active compounds by using surfactant molecules [41]. Regarding the surfactant volume, enhancing the concentration of Tween 80 showed lower PDI values (*P* < 0.05).

As seen in Fig. 2c–d, the PDI value was decreased when the volume of Tween 80 was higher than 19% w/w, while the CSO percentage was less than 6% w/w. Additionally, some lower volumes of Tween 80 produced quite polydispersed NEs with PDI values greater than 0.5. A polydispersity index value lower than 0.2 is ideal, indicating a narrow range of size distribution. The results showed that NEs containing higher than 19% w/w Tween 80 appeared to be monodispersed, while emulsions comprising higher than 17% w/w Tween 80 were moderately polydisperse. The results also show that by increasing the amount of HPW in the nanoemulsion system by more than 71%, the PDI drift was raised.

The turbidity of nanoemulsions tends to increase with increasing refractive index constant, increasing particle concentration and having a maximum value at a particle size where light scattering is strongest [42]. The turbidity was reduced along with the increase of the surfactant content (Fig. 2e–f). Emulsions with the volume of surfactant lower than 19% w/w emerged to be visibly turbid, whereas the nanoemulsions containing 12% and higher Tween 80 were transparent. For NEs, the turbidity increases almost linearly with growing droplet concentration for dilute systems [43]. In general, emulsions with low turbidity had small particle sizes and low PDI.

After optimisation of the model for emulsifying conditions, the optimum parameters for preparation of CSONE were 7.31% of CSO, 19.39% of Tween 80, 73.28% of HPW and 20 min homogenisation time. The experimental results for CSONE particle size, PDI value and turbidity also confirmed the validity of the suggested model. No phase separation was recognised during storage and centrifugation, proving that the formulated nanoemulsion was stable [44].

For a nanoemulsion to be used as a therapeutic application, its formulation must be present at a safe level of cell viability. Cell viability for CSONE was higher than CSO, which may have been caused by the small NE size. Consequently, we decided to use CSO and CSONE at the lowest but most efficient concentration in the subsequent wound healing tests from the cell viability results. Cell viability may be negatively influenced by exposure to bioactive particles and the delivery vehicle, affecting the wound-healing process. Tween surfactants can reduce cell viability by interacting with or entering the cell membrane, and at high concentrations, leads to solubilisation of the membrane [45]. The concentration of Tween 80 in the highest cell viability CSONE was 1.2%, and the cell viability test with Tween 80 alone indicated very small cytotoxicity (less than 9%) at this concentration.

The DPPH assay is based on the hypothesis that a hydrogen donor is an antioxidant and measures the capacity of a probable antioxidant compound to reduce the stable DPPH and its violet colour fades [46]. Our GC-MS profiling revealed oleic acid as one of the major phytoconstituents. According to the literature [47, 48], oleic acid in CSO, a triterpenoid compound, acts as a potent antioxidant. The antioxidant activity in CSO protects skin cells from damage by reactive oxygen species (ROS) and other oxidative adversaries. ROS are central to all wound healing processes since low concentrations of ROS generation are needed to resist invading microorganisms and cell growth signalling [49]. Also, a larger surface area in NE formulation is followed by smaller droplet particles, producing more susceptibility to oxidation due to greater interactions with oxygen, free radicals, and metal peroxides, leading to a higher wound healing efficacy [50].

Wound closure is a potent mechanism that includes the proliferation and migration of skin cells at the wound site. BSR cells (BHK-21cells) used in this study are fibroblast cells used in numerous in vitro assays and are the predominant cell type in the skin [51, 52]. These potential wound healing properties of CSO and CSONE were possibly due to certain enhancements in the proliferation of epithelial cells [53]. Wound closure in CSONE was higher than in CSO at equivalent concentrations, representing a potential enhancement due to nanometre-sized droplets stabilised by surfactants. A higher wound closure rate depends on cell viability [33], and the cell viability of the CSONE was higher than CSO by a factor of 4.2 at 9.12 μg/mL and a factor of 1.1 at 4.56 *μ*g/mL CSO concentrations.

Free fatty acids (FFA) available in CSO, predominantly monounsaturated FFAs such as oleic acid, can obstruct the skin barrier and act as a penetrable enhancer for other compounds present in CSO. In an in vivo study by Bhattacharjee et al. [47], pre-treatment of rats with oleic acid indicated protection against cadmium-induced injuries through multiple mechanisms. Poly and monounsaturated fatty acids are known to influence inflammatory responses, both as soluble, lipoic agents and in the form of phospholipids attached to the cell membrane. For example, topical applications of linolenic and linoleic can modulate the closure of surgically-caused skin wounds [54].

Nguyen et al. [12] reported that the topical application of Calophyllolide (CP), a major component isolated from *C. inophyllum* seeds, can inhibit collagen development and boost wound closure in animal models. By inhibiting myeloperoxidase activity and inducing M2-relation gene expression leading to M2 macrophage skewing, Calophyllolide decreased fibrosis formation and stimulated the closure of the wound area with the epidermis and dermal layers completely formed in 14 days of treatment. In a study on the skin-active effect of *C. inophyllum* oil extract on human keratinocytes and dermal fibroblasts, the mechanism of wound healing was reported based on the up-regulation of genes involved in cell adhesion, cell proliferation and O-glycan synthesis [10]. Although the mechanisms involved in this study are unknown, the wound healing test against BSR cells confirmed their finding with CSO and verified that CSO as NE enhances this effect. CSONE showed 20% higher wound healing than CSO alone at the same concentration. Further investigation is needed for a better understanding of the mechanism of actions of CSONE on wound healing.

## Conclusion

Our study carried out the preparation, characterisation, and application of optimised *Calophyllum inophyllum* seed oil nanoemulsion. The CSONE was prepared using high-shear homogeniser techniques. The optimised CSONE with the droplet size of 45–50 nm was prepared from a mixture of CSO, Tween 80 and HPW. This nanoemulsion was stable, and its size and features remained stable after storage for up to 4 weeks. The optimised nanoemulsion was used for the biological investigation. The analysis of the antioxidant activity of CSO alone, or as NEs, confirms that the structured oil in NEs does not reduce its activity. Our results support the probability of using CSONE as a natural antioxidant in various pharmaceutical areas.

Additionally, cell viability results showed that CSONE is less cytotoxic than CSO on BSR cells at different concentrations. Likewise, the in vitro cell monolayer scratch (wound healing) assay revealed that CSONE with a concentration of CSO as low as 0.4% gave 100% of wound closure after 48 hrs and was comparable with the FGF. The increase in wound closure by CSONE was possible due to the nanoemulsion size of the droplets stabilised by Tween 80 as a surfactant. These findings present that CSONE could be a promising and practical approach to in vitro wound healing by boosting the proliferation and migration of epidermal cells.

## Supplementary Information

**Additional file 1: Table S1.** Mixture components and process factor values used in Optimal Combined Design (OCD). **Table S2.** Mixture components and process factors values used in Optimal Combined Design (OCD). **Figure S1.** The scatter plot of predicted values vs actual values. This plot demonstrated the interaction effect between variables; A: size; B: Polydispersity index (PDI); and C: Turbidity. **Figure S2.** Visual appearance of optimized Calophyllum inophyllum seed oil nanoemulsion (CSONE). **Figure S3.** Wound closure percentage of CSO and CSONE at 24 hr and 48 hr in BSR cells. Calophyllum inophyllum seed oil (CSO). CSO nanoemulsion (CSONE). CSO1 and CSONE1: 9.12 μg/mL. CSO2 and CSONE2: 4.56 μg/mL. Blank indicates untreated cells (negative control) and FGF indicates Fibroblast Growth Factors (positive control). Each value is the mean of three replicates with standard deviation (± SD) and analysed by Two-way-ANOVA. (*n* = 3; *p* < 0.05).

## Data Availability

The datasets used and/or analysed during the current study are available from the corresponding author upon reasonable request.

## Abbreviations

BSR: Baby hamster kidney a clone BHK-21
CSONE: *Calophyllum inophyllum* seed oil nanoemulsion
CSO: *Calophyllum inophyllum* seed oil
CP: Calophyllolide
DMSO: Dimethyl sulfoxide
DoE: Design of experiments
DPPH: 1,1-diphenyl-2-picrylhydrazyl (DPPH)-2,2-diphenyl-1-picrylhydrazyl
FAME: Fatty acid methyl esters
FFA: Free fatty acids
FGF: Fibroblast growth factor
GC-MS: Gas chromatography/mass spectrometry
HPW: High pure water
LC/MS: Liquid chromatography/mass spectrometry
MTT: 3-(4,5-dimethylthiazol-2-yl)-2,5-diphenyltetrazolium bromide
NE: Nanoemulsion
O/W: Oil-in-water
SC: Stratum Corneum
TEM: Transmission electron microscope
