# Supplementary material for: Optimisation of Calophyllum inophyllum seed oil nanoemulsion as a potential wound healing agent
Source: BMC Complement Med Ther. 2022 Nov 4;22:285. doi: 10.1186/s12906-022-03751-6 (PMC9635111; doi:10.1186/s12906-022-03751-6)
Supplement: Supplementary file 1 — Additional file 1: Table S1. Mixture components and process factor values used in Optimal Combined Design (OCD). Table S2. Mixture components and process factors values used in Optimal Combined Design (OCD). Figure S1. The scatter plot of predicted values vs actual values. This plot demonstrated the interaction effect between variables; A: size; B: Polydispersity index (PDI); and C: Turbidity. Figure S2. Visual appearance of optimized Calophyllum inophyllum seed oil nanoemulsion (CSONE). Figure S3. Wound closure percentage of CSO and CSONE at 24 hr and 48 hr in BSR cells. Calophyllum inophyllum seed oil (CSO). CSO nanoemulsion (CSONE). CSO1 and CSONE1: 9.12 μg/mL. CSO2 and CSONE2: 4.56 μg/mL. Blank indicates untreated cells (negative control) and FGF indicates Fibroblast Growth Factors (positive control). Each value is the mean of three replicates with standard deviation (± SD) and analysed by Two-way-ANOVA. (n = 3; p < 0.05). [file 12906_2022_3751_MOESM1_ESM.docx]

Supplementary Materials for “Optimization of *Calophyllum inophyllum* seed oil nanoemulsion as a potential wound healing agent”

Elnaz Saki^a^, Vinuthaa Murthy^a^, Richard Weir^b^, Roshanak Khandanlou^a^, Hao Wang^a^, Jo Wapling^c^

^a^ College of Engineering, IT & Environment, Charles Darwin University, Australia

^b^ Berrimah Veterinary Laboratory, Department of Industry, Tourism and Trade Northern Territory Government, Australia

^c^ Menzies School of Health Research, Charles Darwin University, Australia

Table S1:Mixture components and process factor values used in Optimal Combined Design (OCD).

| Factors | Name | Units | Type | Minimum | Maximum | Coded Low | Coded High |
| --- | --- | --- | --- | --- | --- | --- | --- |
| A: X_1_ | CSO | % | Mixture | 4 | 8 | +0 ↔ 4 | +0.16 ↔ 8 |
| B: X_2_ | Tween 80 | % | Mixture | 3 | 24 | +0 ↔ 3 | +0.84 ↔ 24 |
| C: X_3_ | HPW | % | Mixture | 68 | 93 | +0 ↔ 68 | +1 ↔ 93 |
|  |  |  |  | Total = 100 | |  |  |
| D: X_4_ | Time | min | Numeric | 10.00 | 40.00 | -1 ↔ 10.00 | +1 ↔ 40.00 |

Notes: CSO (X1), Tween 80 (X2), HPW (X3) and homogenisation time (X4).

Table S2: Mixture components and process factors values used in Optimal Combined Design (OCD).

| Run | A: CSO  (%) | B: Tween 80  (%) | C: HPW  (%) | D: Time  (min) | Droplet Size  (nm) | | PDI | Turbidity |
| --- | --- | --- | --- | --- | --- | --- | --- | --- |
| 1 | 5.7 | 15.5 | 78.7 | 10.0 | 70.00 | 0.28 | | 61.90 |
| 2 | 6.4 | 3.0 | 90.6 | 10.0 | 158.66 | 1.09 | | 3018.03 |
| 3 | 4.0 | 24.0 | 72.0 | 38.5 | 44.36 | 0.53 | | 1.15 |
| 4 | 4.0 | 6.7 | 89.2 | 10.0 | 108.70 | 0.67 | | 2138.33 |
| 5 | 4.0 | 24.0 | 72.0 | 11.5 | 16.26 | 0.36 | | 107.87 |
| 6 | 8.0 | 17.1 | 74.8 | 17.8 | 77.90 | 0.31 | | 116.28 |
| 7 | 8. | 20.8 | 71.1 | 25.1 | 34.63 | 0.20 | | 20.67 |
| 8 | 5.5 | 24.0 | 70.4 | 25.0 | 19.10 | 0.16 | | 14.22 |
| 9 | 8.0 | 12.4 | 79.5 | 40.0 | 101.80 | 0.46 | | 689.10 |
| 10 | 8.0 | 3.0 | 89.0 | 25.0 | 141.56 | 0.81 | | 3154.51 |
| 11 | 4.6 | 3.0 | 92.3 | 25.0 | 120.63 | 0.64 | | 2654.74 |
| 12 | 5.6 | 14.2 | 80.2 | 40.0 | 86.63 | 0.22 | | 78.05 |
| 13 | 8.0 | 9.0 | 82.9 | 33.1 | 104.66 | 0.56 | | 2579.81 |
| 14 | 6.2 | 12.1 | 81.5 | 25.0 | 79.24 | 0.42 | | 290.32 |
| 15 | 4.0 | 4.8 | 91.1 | 40.0 | 134.50 | 0.81 | | 1664.77 |
| 16 | 8.0 | 18.1 | 73.9 | 32.3 | 104.93 | 0.32 | | 124.99 |
| 17 | 7.6 | 24.0 | 68.3 | 10.0 | 56.20 | 0.19 | | 87.64 |
| 18 | 4.0 | 3.0 | 93.0 | 32.5 | 119.30 | 0.67 | | 2586.52 |
| 19 | 7.0 | 24.0 | 68.9 | 40.0 | 48.47 | 0.29 | | 22.72 |
| 20 | 4.0 | 16.5 | 79.4 | 25.0 | 36.77 | 0.46 | | 10.33 |
| 21 | 8.0 | 10.0 | 81.9 | 10.0 | 130.82 | 0.78 | | 2893.90 |
| 22 | 6.2 | 12.1 | 81.5 | 25.0 | 81.23 | 0.41 | | 354.64 |
| 23 | 6.8 | 3.0 | 90.1 | 40.0 | 112.5 | 0.52 | | 2639.49 |
| 24 | 4.0 | 3.0 | 93.0 | 17.3 | 156.07 | 1.00 | | 2649.53 |


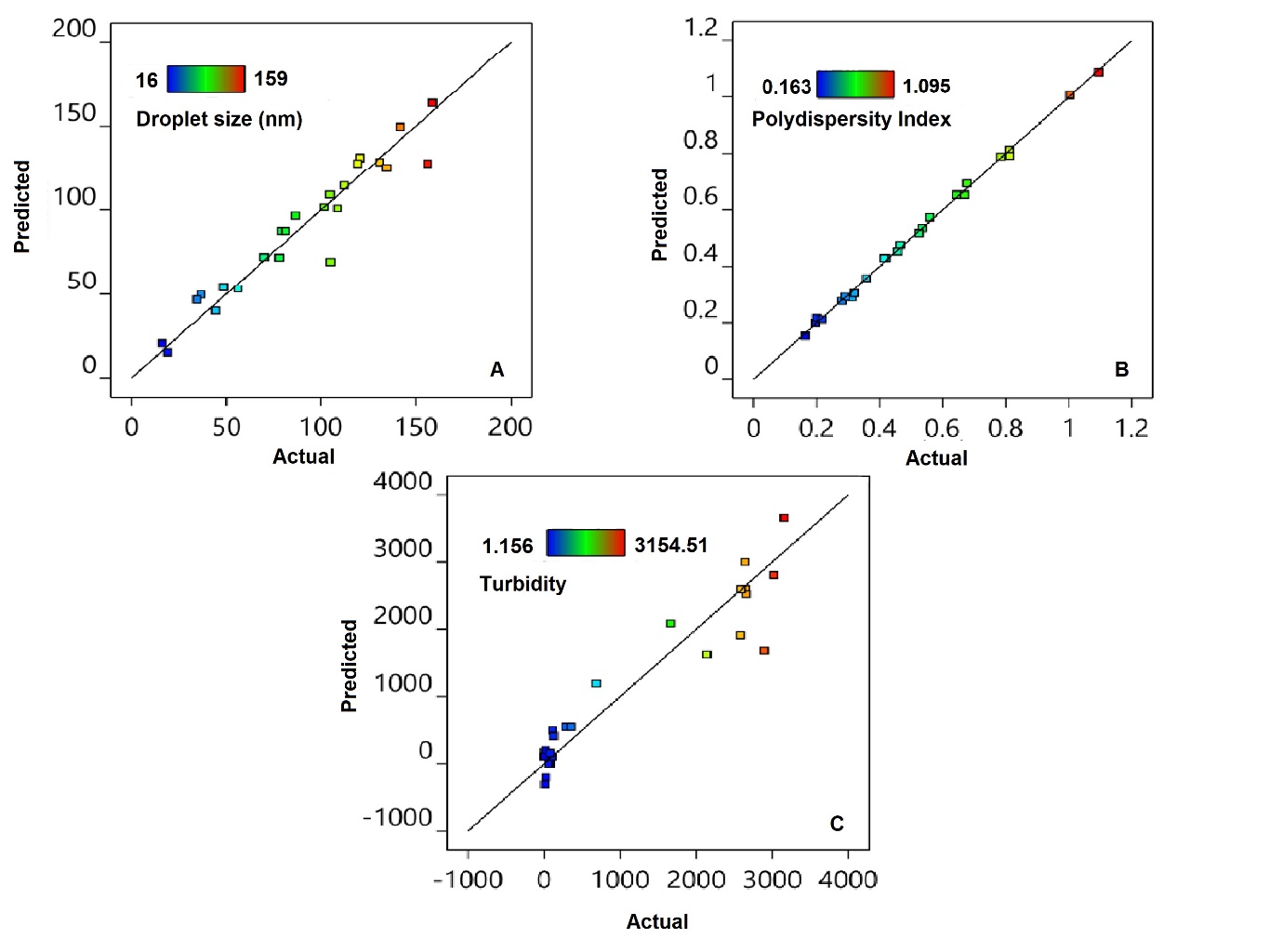


Figure S1: The scatter plot of predicted values vs actual values. This plot demonstrated the interaction effect between variables; A: size; B: Polydispersity index (PDI); and C: Turbidity.


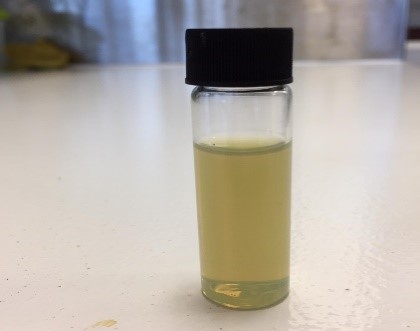


Figure S2: Visual appearance of optimized Calophyllum inophyllum seed oil nanoemulsion (CSONE).


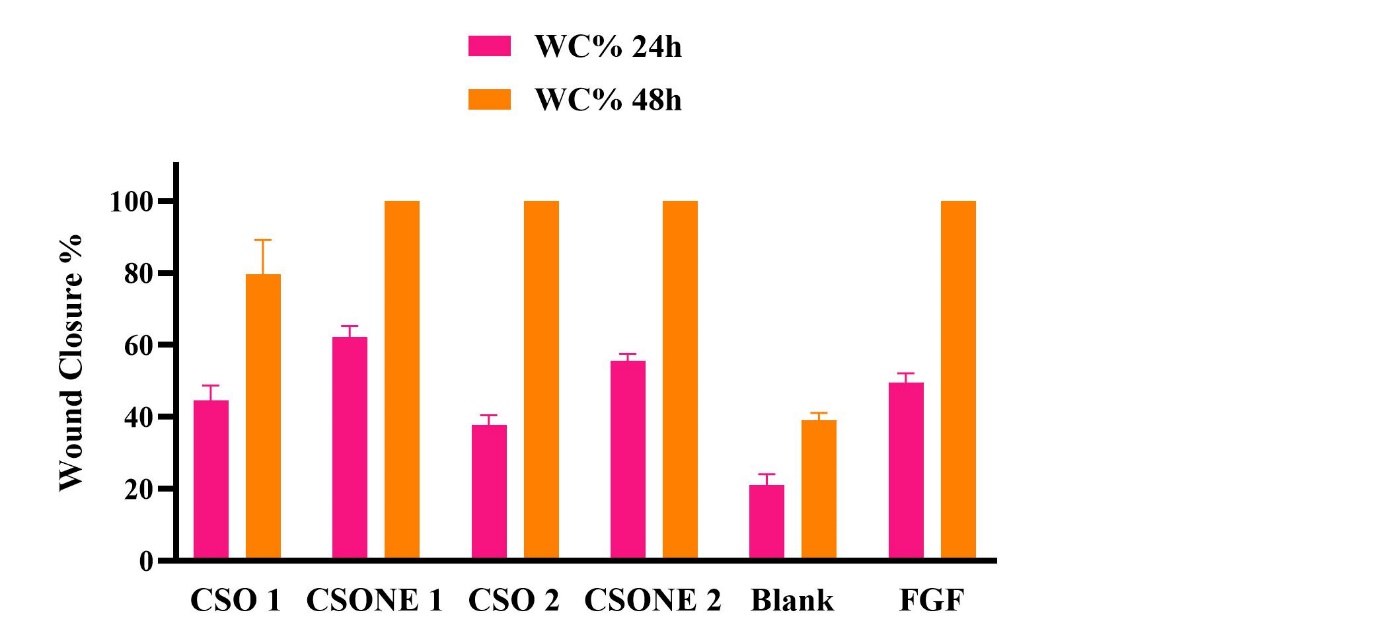


Figure S3: Wound closure percentage of CSO and CSONE at 24 hr and 48 hr in BSR cells. Calophyllum inophyllum seed oil (CSO). CSO nanoemulsion (CSONE). CSO1 and CSONE1: 9.12 µg/mL. CSO2 and CSONE2: 4.56 µg/mL. Blank indicates untreated cells (negative control) and FGF indicates Fibroblast Growth Factors (positive control). Each value is the mean of three replicates with standard deviation (± SD) and analysed by Two-way-ANOVA. (n = 3; p < 0.05).
